# Comorbidity in Patients with Idiopathic Pulmonary Fibrosis: Evaluation Using the Charlson, TORVAN and GAP Indices

**DOI:** 10.3390/jcm15093421

**Published:** 2026-04-29

**Authors:** Soledad Torres Tienza, Javier de Miguel-Díez, Carlos Gutiérrez Ortega, José Javier Jareño Esteban

**Affiliations:** 1 Department of Pulmonology, Hospital Central de la Defensa “Gómez Ulla”, 28047 Madrid, Spain; soledadtorrestienza@gmail.com (S.T.T.); jjjarenoesteban@yahoo.es (J.J.J.E.); 2 Faculty of Medicine, Universidad Complutense de Madrid, 28040 Madrid, Spain; 3 Respiratory Department, Hospital General Universitario Gregorio Marañón, 28007 Madrid, Spain; 4 Gregorio Marañón Biomedical Research Insitute (IiSGM), 28007 Madrid, Spain; 5 Department of Preventive Medicine, Hospital Central de la Defensa “Gómez Ulla”, 28047 Madrid, Spain; kargut13@gmail.com

**Keywords:** IPF, comorbidity, Charlson index, GAP, TORVAN, survival

## Abstract

**Introduction**: Idiopathic pulmonary fibrosis (IPF) is associated with high morbidity and mortality and a substantial burden of comorbidities, which may influence prognosis and survival. This study aimed to evaluate the burden of comorbidity in patients with IPF receiving antifibrotic therapy using the Charlson, TORVAN, and GAP indices and to analyse their relationships and prognostic impact on survival. **Methods**: Retrospective observational study including patients with IPF diagnosed according to ATS/ERS/JRS/ALAT criteria. Patients receiving antifibrotic therapy between June 2010 and September 2025 were included. Baseline comorbidities were recorded, and the Charlson, TORVAN, and GAP indices were calculated. Associations between indices were assessed using chi-square tests and kappa statistics. Survival was analysed using Kaplan–Meier curves and compared with the log-rank test. Cox proportional hazards regression and model comparison metrics (Harrell’s C-index and Akaike Information Criterion) were also performed to assess the independent prognostic value of each index. **Results**: Seventy-two patients were included (76.7% male; mean age 73.8 ± 7.4 years). Pirfenidone was prescribed in 63.9% and nintedanib in 36.1%. The most frequent comorbidities were gastro-oesophageal reflux disease (62.5%), arterial hypertension (57.5%), pulmonary hypertension (32.9%), diabetes mellitus (24.7%), and non-metastatic solid tumours (17.6%), including lung cancer. Survival differed significantly according to GAP stage (*p* = 0.020) and Charlson categories (*p* = 0.006). The TORVAN stage was associated with the GAP stage (*p* < 0.001; kappa = 0.246), whereas the Charlson index showed no association with GAP or TORVAN. **Conclusions**: In this cohort of patients with IPF receiving antifibrotic therapy, both the GAP and Charlson indices were associated with survival. These findings suggest that combining disease-specific and comorbidity indices may provide a more comprehensive prognostic assessment, although further validation in larger cohorts is required.

## 1. Introduction

Idiopathic pulmonary fibrosis (IPF) is a chronic, progressive interstitial lung disease with a poor prognosis, whose pathogenesis is considered multifactorial, arising from the interaction between genetic susceptibility and environmental exposures. The disease is characterized by progressive fibrotic remodelling of lung parenchyma, leading to irreversible decline in pulmonary function, worsening dyspnoea, and ultimately respiratory failure. Despite advances in diagnosis and management, IPF remains associated with high morbidity and mortality worldwide [1,2].

Recent epidemiological studies have highlighted the substantial mortality associated with IPF across Europe, with some of the highest rates reported in countries such as Ireland, the United Kingdom, Finland, Sweden, Luxembourg, and Spain, expressed per 100,000 inhabitants per year [3].

The introduction of antifibrotic therapies, including pirfenidone and nintedanib, has represented an important advance in the management of the disease. These treatments have been shown to slow the decline in lung function and may contribute to improving survival in selected patient populations [3,4].

Despite these therapeutic advances, comorbidities are highly prevalent in patients with IPF and may significantly influence disease course and survival [5,6,7,8,9,10]. Their systematic identification and evaluation have gained increasing relevance, as they may affect clinical management and improve risk stratification [8,9]. Several studies have shown that conditions such as cardiovascular disease, pulmonary hypertension, gastro-oesophageal reflux disease, metabolic disorders, and malignancies are frequently present in patients with IPF and may affect both quality of life and clinical outcomes. Therefore, a comprehensive assessment of comorbidity burden may provide additional prognostic information beyond traditional physiological parameters [5,6,7,8,9,10].

Beyond their prevalence, increasing attention has been paid in recent years to the impact of comorbidities on the clinical course of IPF. Several observational studies have shown that cardiovascular diseases, metabolic disorders, pulmonary hypertension, and gastro-oesophageal reflux disease are highly prevalent in patients with IPF and may influence disease progression, quality of life, and survival. The systematic assessment of comorbidity burden has therefore become an important component of patient evaluation, particularly in the era of antifibrotic therapies, where longer survival allows the clinical impact of comorbid conditions to become more evident [6,8,10].

To enhance prognostic assessment in IPF, several indices have been developed. The GAP index is widely used for risk stratification based on clinical and functional variables [11]. The Charlson index allows standardized quantification of global comorbidity burden [12]. More recently, the TORVAN scale has been proposed as a prognostic model integrating clinical variables, lung function parameters, and selected comorbidities to improve survival prediction in IPF. However, its external validation and applicability in real-world cohorts, particularly in patients receiving antifibrotic therapy, remain limited [13,14].

Given the increasing recognition of the role of comorbidities in IPF, a better understanding of their relationship with established prognostic indices may contribute to a more comprehensive evaluation of disease severity and patient outcomes. In addition, the management of patients with IPF increasingly relies on multidisciplinary interstitial lung disease (ILD) units, where pulmonologists, radiologists, and pathologists collaborate to establish diagnosis, optimize treatment, and monitor disease progression. Within this context, the integration of clinical prognostic indices and comorbidity assessment may help improve risk stratification and guide clinical decision-making in routine practice [1,2].

In addition to their prognostic implications, comorbidities may also influence the clinical management of patients with IPF. Many of these conditions require specific diagnostic evaluation and treatment strategies that may interact with the management of the underlying fibrotic disease. For instance, cardiovascular diseases, pulmonary hypertension, and metabolic disorders may require coordinated management involving multiple specialties. As a consequence, the evaluation of comorbidity burden has progressively gained relevance in the routine assessment of patients with IPF. Identifying these conditions may not only improve prognostic stratification but also facilitate a more comprehensive approach to patient care within multidisciplinary interstitial lung disease units [5,6,7,8,9,10].

The aim of this study was to evaluate the burden of comorbidity in patients with IPF receiving antifibrotic therapy and to assess the prognostic performance of the Charlson, GAP, and TORVAN indices and their association with survival.

## 2. Materials and Methods

This retrospective observational study included patients with a diagnosis of IPF established according to the ATS/ERS/JRS/ALAT clinical practice guidelines published in 2018 and subsequently updated in 2022 [1,2]. Patients were recruited from the southern area of Madrid, and the diagnosis was confirmed within a multidisciplinary interstitial lung disease unit accredited by the Spanish Society of Pulmonology and Thoracic Surgery (SEPAR) in 2023. Within this multidisciplinary framework, diagnostic evaluation included clinical assessment, high-resolution computed tomography, and, when required, histopathological evaluation, with the final diagnosis established through multidisciplinary discussion.

The inclusion period ranged from 1 June 2010 to 30 September 2025. A total of 72 patients receiving antifibrotic therapy were included. Treatment decisions were made according to standard clinical practice and international clinical guidelines during the study period [1,2].

The study was approved by the Ethics Committee of Hospital Central de la Defensa “Gómez Ulla” and was conducted in accordance with the principles of the Declaration of Helsinki and its subsequent revisions. Given the retrospective nature of the study and the use of anonymized data, individual informed consent was not required.

Clinical and demographic data were obtained through electronic medical record review. Baseline comorbidities at the time of diagnosis were recorded, together with the variables required for the calculation of the Charlson, TORVAN, and GAP indices [11,12,13,14]. Comorbidities were identified based on documented clinical diagnoses in the patients’ electronic medical records at baseline. The comorbidities included those incorporated into the Charlson Comorbidity Index, as well as other clinically relevant conditions frequently described in patients with IPF. Data collection was performed systematically to ensure consistent identification of comorbid conditions across the study cohort.

All clinical data were reviewed independently by the investigators using standardized criteria in order to ensure consistency in the identification of comorbid conditions. When available, complementary diagnostic tests, specialist reports, and imaging findings were reviewed to confirm the presence of relevant comorbidities. Lung function parameters used for the calculation of the GAP index were obtained from pulmonary function tests performed at the time of diagnosis or within the first months of clinical evaluation. The TORVAN index was calculated according to the variables originally described in the model, integrating clinical characteristics, lung function parameters, and selected comorbidities.

Pulmonary hypertension was defined according to the right heart catheterization when available or echocardiographic findings compatible with pulmonary hypertension. Gastro-oesophageal reflux disease was defined based on documented clinical diagnosis, compatible symptoms, and/or treatment with proton pump inhibitors, and in some cases, it was supported by complementary diagnostic tests.

All 72 patients included in the study had complete data for the variables required for the calculation of the Charlson, GAP, and TORVAN indices. No imputation methods were used. Lung function parameters required for the GAP index were available for all patients from pulmonary function tests performed at the time of diagnosis or during the initial clinical evaluation.

Statistical analyses were performed using IBM SPSS Statistics version 27.0 (IBM Corp., Armonk, NY, USA). Quantitative variables were expressed as mean ± standard deviation or median (interquartile range), depending on the distribution. Qualitative variables were presented as absolute frequencies and percentages.

Agreement and association between index categories were assessed using Cohen’s kappa coefficient and the chi-square test, respectively. Survival was estimated using Kaplan–Meier curves and compared between groups using the log-rank (Mantel–Cox) test. Kaplan–Meier plots were truncated at 10 years of follow-up for visual clarity, while all available follow-up data were included in the statistical analysis.

To further assess the independent prognostic value of each index, univariable and multivariable Cox proportional hazards regression analyses were performed. In the univariable analysis, each index and clinical variable was entered separately as a predictor of all-cause mortality. In the multivariable analysis, each index was included in separate models adjusted for age, sex, FVC%, and antifibrotic treatment. DLCO % was not included in the multivariable models due to its correlation with FVC% and to limit the number of covariates relative to the number of events. In addition, given that lung function parameters are already incorporated into the GAP and TORVAN indices, the multivariable models should be interpreted with caution due to potential collinearity. The discriminative ability of each model was evaluated using Harrell’s C-index, with values closer to 1.0 indicating better discrimination. Model fit was compared using the Akaike Information Criterion (AIC), with lower values indicating a better fit. A *p* value < 0.05 was considered statistically significant.

## 3. Results

### 3.1. Baseline Characteristics of the Cohort

Seventy-two patients with IPF receiving antifibrotic treatment were included in the study. The cohort was predominantly elderly and male, with preserved lung volumes and a moderately reduced diffusing capacity. Most patients were treated with pirfenidone, while the remainder received nintedanib. Baseline clinical and functional characteristics are summarized in Table 1.

### 3.2. Comorbidities in the Cohort

Comorbidities were common in the cohort. Gastro-oesophageal reflux disease and arterial hypertension were the most frequent conditions, followed by pulmonary hypertension and diabetes mellitus (Table 2).

Cardiovascular comorbidities were common, including heart failure and arrhythmias. A relevant proportion of patients also presented oncological comorbidities, mainly non-metastatic solid tumours. Respiratory comorbidities such as chronic obstructive pulmonary disease were also observed.

Overall, the comorbidity profile suggests a substantial burden of cardiovascular, metabolic, and respiratory conditions in patients with IPF (Table 2).

### 3.3. Comorbidity and Prognostic Indices

According to the Charlson index, most patients were classified in the low-risk group, with fewer patients in the intermediate- and high-risk categories (Table 3).

For the GAP index, more than half of the patients were in stage I, followed by stage II, while stage III represented a minority.

Patients classified according to the TORVAN scale were distributed across all stages, with a predominance of stages I and III.

### 3.4. Association Between Comorbidity and Prognostic Indices

The association between index categories was evaluated using chi-square tests and agreement with the kappa coefficient. A significant association was observed between the TORVAN scale and the GAP stage (χ^2^ = 32.705; df = 6; *p* < 0.001), with low-to-moderate agreement (Kappa = 0.246; *p* < 0.001). Descriptively, patients classified as TORVAN I were mostly concentrated in GAP I, whereas TORVAN stages III–IV were associated with a higher proportion of patients in GAP II–III, showing a distribution consistent with more advanced prognostic categories.

In contrast, no statistically significant associations were identified between the Charlson index and GAP stage (χ^2^ = 1.778; df = 4; *p* = 0.777; Kappa = −0.052; *p* = 0.512) nor between the Charlson index and the TORVAN scale (χ^2^ = 5.807; df = 6; *p* = 0.445; Kappa = −0.006; *p* = 0.921). Full results are presented in Table 4.

### 3.5. Survival

Of the 72 patients included, 46 (63.9%) received pirfenidone, and 26 (36.1%) received nintedanib. Both treatment groups were comparable at baseline in terms of age, sex, FVC%, GAP stage, and Charlson index (all *p* > 0.05), with no significant differences in survival between groups (log-rank *p* = 0.740). During follow-up, 34 deaths were recorded (47.2%), while 38 patients (52.8%) were alive at the end of the study period (censored). Median overall survival was 6.6 years (95% CI: 5.5–7.7 years). No patients underwent lung transplantation during the follow-up period.

**Figure 1:**
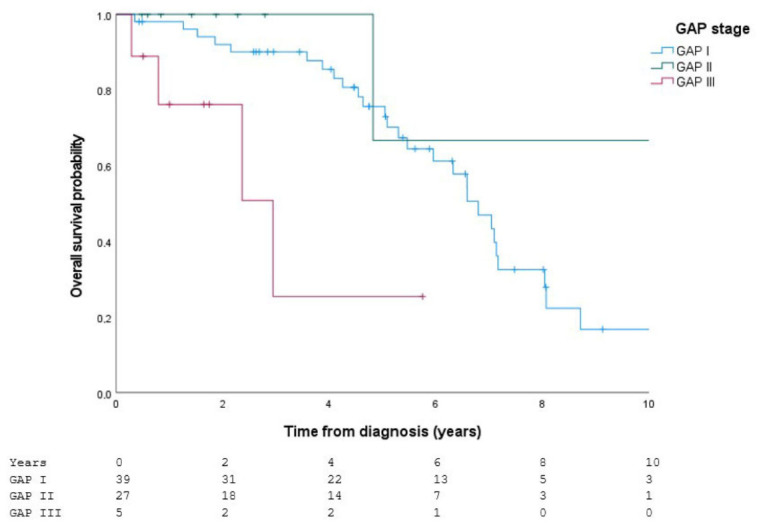
Survival according to the GAP stage. Kaplan–Meier survival curves stratified by GAP stage (I–III) in patients with IPF receiving antifibrotic therapy. Crosses indicate censored observations during follow-up.

When stratified by GAP index, significant differences in survival were observed between stages (log-rank: χ^2^ = 7.794; df = 2; *p* = 0.020), with lower survival observed in more advanced stages (Figure 1).

**Figure 2:**
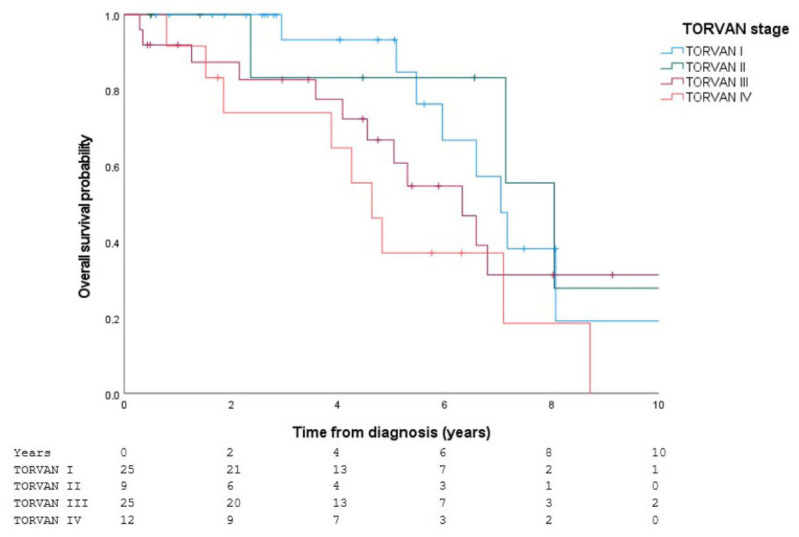
Survival according to the TORVAN stage. Kaplan–Meier survival curves stratified by TORVAN stage (I–IV) in patients with IPF receiving antifibrotic therapy. Crosses indicate censored observations during follow-up.

For the TORVAN scale, no statistically significant differences were observed among the four stages (log-rank: χ^2^ = 5.137; df = 3; *p* = 0.162) (Figure 2).

**Figure 3:**
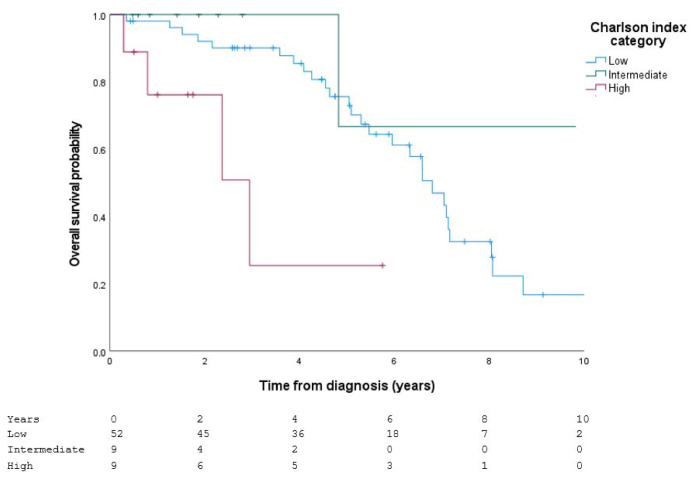
Survival according to the Charlson Comorbidity Index categories. Kaplan–Meier survival curves stratified by baseline Charlson Comorbidity Index categories (low, intermediate, and high) in patients with IPF receiving antifibrotic therapy. Crosses indicate censored observations during follow-up.

The Charlson index, categorized into low, intermediate, and high risk, showed significant survival differences (log-rank: χ^2^ = 10.076; df = 2; *p* = 0.006), with lower survival in the high-risk group (Figure 3).

Descriptively, median survival appeared to be shorter in the high Charlson group compared with the most advanced GAP and TORVAN stages; however, as the subgroups across indices are defined by different criteria and do not represent the same patients, direct comparisons should be interpreted with caution. A more appropriate comparison of the prognostic performance of the three indices is provided by the model-based analyses presented in Section 3.6 (Table 5).

### 3.6. Cox Regression Analysis and Model Comparison

In the univariable analysis, GAP stage, TORVAN stage, FVC%, and DLCO% were significant predictors of all-cause mortality, while the Charlson index did not reach statistical significance in the univariable model. Results are presented in Table 6.

In multivariable Cox models adjusted for age, sex, FVC%, and antifibrotic treatment, none of the indices retained independent statistical significance. This attenuation is consistent with the potential collinearity introduced by including FVC% as a covariate, given that this parameter is already incorporated in the calculation of GAP and TORVAN.

The three models showed similar discriminative ability, with Harrell’s C-index values ranging from 0.682 to 0.692 and comparable AIC values (Table 7), indicating that no single index provided a substantially better model fit.

## 4. Discussion

In this study, comorbidity in patients with IPF receiving antifibrotic therapy was comprehensively assessed using the Charlson Comorbidity Index and its relationship with two IPF-specific prognostic indices, GAP and TORVAN, as well as their impact on survival.

The clinical relevance of comorbidity in IPF has gained increasing attention in recent years. As antifibrotic therapies have modified the natural history of the disease and prolonged survival, the relative impact of comorbid conditions on prognosis and patient management has become more apparent. In this context, the systematic evaluation of comorbidity burden may provide valuable complementary information to disease-specific indices traditionally used in IPF. Several studies have highlighted that cardiovascular disease, metabolic disorders, and pulmonary hypertension are among the most frequent comorbidities and may significantly influence outcomes in this population [6,8,15].

The integration of comorbidity assessment into routine clinical evaluation may therefore contribute to a more comprehensive understanding of disease severity. In multidisciplinary interstitial lung disease units, where pulmonologists, radiologists, and other specialists collaborate in the management of these patients, the consideration of comorbid conditions may support more individualized clinical decision-making and follow-up strategies.

The relevance of comorbidity in IPF has been widely described in the literature. Systematic reviews have highlighted the high burden and heterogeneity of comorbidities in these patients, as well as their potential clinical and prognostic implications [5]. Previous observational studies have also shown that certain comorbidities are independently associated with increased mortality in IPF [7,15]. Our findings suggest that comorbidity is common in this population and that the different indices may provide complementary, rather than interchangeable, prognostic information [3,6,8,10,16].

The Charlson index showed that most patients had a low global comorbidity burden, although a relevant subgroup was classified as having intermediate or high risk. This observation is consistent with other IPF cohorts, in which cardiovascular and metabolic comorbidities are frequent but do not always translate into high Charlson scores [7,16]. In our cohort, the Charlson index was significantly associated with survival, suggesting its potential value as a tool for global prognostic stratification in patients with IPF receiving antifibrotic treatment [12,17].

Cox regression analysis identified the prognostic value of GAP and TORVAN stages in the univariable analysis. In the multivariable models, none of the indices retained independent statistical significance after adjustment, consistent with the structural collinearity between FVC% and the disease-specific indices and with the limited statistical power given the sample size. All three indices showed similar discriminative ability, suggesting that no single index appears to be clearly superior to the others.

The GAP index, considered the classical prognostic standard in IPF, demonstrated an adequate ability to discriminate survival in our cohort, with a progressive reduction in survival as the stage increased. These results are consistent with the original validation studies and with subsequent analyses in real-world cohorts, supporting the external validity of GAP in our clinical setting [11,18,19]. Similar findings regarding stage-related survival in patients with IPF receiving antifibrotic therapy have also been reported in recent studies [20].

Regarding the TORVAN scale, the distribution of patients across stages was heterogeneous. Although statistically significant differences in survival between TORVAN stages were not observed in our cohort, this finding may be partly explained by the limited sample size and the relatively small number of patients in some subgroups, which may reduce the statistical power to detect significant differences [13,14]. Furthermore, the structural overlap between TORVAN and GAP (both of which incorporate lung function parameters) may limit the ability of TORVAN to provide independent prognostic discrimination in this cohort.

An important aspect of this study is the direct comparison between the different indices. A significant association was observed between TORVAN stage and GAP stage, although concordance was only low to moderate, suggesting that both indices capture related but not completely overlapping prognostic dimensions [14]. In contrast, no significant association was found between the Charlson index and either TORVAN or GAP, indicating that global comorbidity burden, as measured by the Charlson index, provides distinct and complementary information to that obtained from IPF-specific indices [7,15].

The analysis of comorbidities revealed a high prevalence of gastro-oesophageal reflux disease, arterial hypertension, diabetes mellitus, and pulmonary hypertension, findings consistent with previous reports [6,21]. A relevant burden of cardiovascular comorbidities was also observed, together with a non-negligible prevalence of solid tumours, including lung cancer [22]. These findings reinforce the need for a comprehensive assessment of patients with IPF beyond lung function and disease-specific prognostic indices [6,15].

From a clinical perspective, the integration of comorbidity assessment into the routine evaluation of patients with IPF may provide relevant information for clinical decision-making. The coexistence of multiple comorbid conditions often reflects the complex systemic profile of these patients and may influence therapeutic strategies, follow-up intensity, and overall prognosis. In this context, indices that quantify global comorbidity burden may complement disease-specific prognostic tools and contribute to a more comprehensive risk assessment.

In addition, the increasing recognition of the systemic nature of IPF highlights the importance of comprehensive patient evaluation in routine clinical practice. The identification and appropriate management of comorbidities may contribute to improved patient monitoring and may help optimize therapeutic strategies in this complex patient population. Future research should aim to explore whether integrated prognostic models combining disease severity and comorbidity burden may improve risk stratification and potentially guide personalized management strategies in patients with IPF [15,21].

This study has several limitations. First, its retrospective design may be associated with biases in data collection. Second, the relatively small sample size (*n* = 72) represents an inherent constraint of a single-centre retrospective design and limits the statistical power of the multivariable analyses. In Cox regression modelling, the widely cited rule of at least 10 events per variable (EPVs) suggests that our models may be underpowered to detect independent effects of moderate magnitude. This may explain, at least in part, the loss of statistical significance observed for the prognostic indices in the multivariable models compared to the univariable analyses. Furthermore, the small number of patients in the highest-risk subgroups—particularly GAP Stage III (*n* = 5)—results in wide confidence intervals that should be interpreted with caution. Therefore, the findings of this study should be interpreted as exploratory and hypothesis-generating and require confirmation in larger, preferably multicentre, prospective cohorts. Furthermore, this is a single-centre study, which may limit the generalizability of the results to other populations or healthcare settings. Due to the retrospective design and the limited cohort size, a formal a priori power calculation was not performed.

Overall, our findings suggest that both the Charlson index and the GAP index may offer additional prognostic information regarding survival in patients with IPF receiving antifibrotic therapy. In our cohort, the Charlson index showed a significant association with survival, reinforcing its usefulness as a tool for global comorbidity assessment [12,15]. The TORVAN scale was significantly associated with GAP stage, indicating that both indices share prognostic dimensions, although no significant survival differences between TORVAN stages were observed [14]. These results suggest that, in our cohort, the TORVAN scale did not provide additional prognostic discrimination beyond classical indices, although it may represent a complementary tool when used in combination with other indices. Multicentre studies with larger sample sizes are needed to better define its role in prognostic stratification in IPF.

These findings may have relevant implications for clinical practice. The systematic evaluation of comorbidity burden in patients with IPF may complement disease-specific prognostic indices and contribute to a more comprehensive assessment of patient risk. In clinical settings, particularly within multidisciplinary interstitial lung disease units, the integration of global comorbidity indices such as the Charlson score together with disease-specific indices (GAP and TORVAN) may support more individualized risk stratification and follow-up strategies [15,21]. Future research should focus on validating these observations in larger multicentre cohorts and exploring whether integrated prognostic models combining comorbidity burden and disease severity could improve prognostic assessment and clinical decision-making in patients with IPF.

Overall, these results support the importance of considering both disease-specific severity and global comorbidity burden when evaluating patients with IPF.

## 5. Conclusions

In this cohort of patients with IPF receiving antifibrotic therapy, comorbidity was frequent and appeared to be associated with prognosis and survival. The Charlson Comorbidity Index appeared to be useful as a global tool for assessing overall comorbidity burden, potentially providing additional prognostic information beyond disease-specific indices.

The GAP index appeared to retain its ability to discriminate survival across stages, supporting its role as a disease-specific prognostic index in IPF. The TORVAN index was associated with GAP stage, suggesting that both indices capture prognostic dimensions intrinsic to IPF, although no significant survival differences between TORVAN stages were observed in our cohort. In multivariable analyses, none of the indices retained independent statistical significance after adjustment, consistent with the limited sample size and the structural collinearity between FVC% and the disease-specific indices.

Overall, the integrated assessment of global comorbidity using the Charlson index together with IPF-specific prognostic indices (TORVAN and GAP) may contribute to a more comprehensive stratification, although these findings require confirmation in larger cohorts of patients with IPF in clinical practice.

## Figures and Tables

**Table 1:** Baseline characteristics of the study cohort.

Variable	Results (*n* = 72)
Age at diagnosis, years (mean ± SD)	73.8 ± 7.4
Male sex	55 (76%)
Female sex	17 (24%)
Forced vital capacity, % predicted (mean ± SD)	87.4 ± 22.7
FEV_1_, % predicted (mean ± SD)	93.1 ± 22.5
DLCO, % predicted (mean ± SD)	63.3 ± 20.8
Antifibrotic treatment	
Pirfenidone	46 (64%)
Nintedanib	26 (36%)

**Table 2:** Comorbidities in the study cohort.

Comorbidity	*n* (%)
Gastro-oesophageal reflux disease	45 (62.5)
Arterial hypertension	42 (57.5)
Pulmonary hypertension	24 (32.9)
Diabetes mellitus	18 (24.7)
Heart failure	12 (17.6)
Non-metastatic solid tumour	12 (17.6)
Arrhythmia	11 (15.7)
Chronic obstructive pulmonary disease	11 (15.5)
Depressive disorder	8 (11.4)

**Table 3:** Distribution of Charlson, GAP, and TORVAN indices.

Index	Category	*n* (%)
Charlson	Low	53 (73.6)
	Intermediate	10 (13.9)
	High	9 (12.5)
GAP	I	40 (55.6)
	II	27 (37.5)
	III	5 (6.9)
TORVAN	I	26 (36.1)
	II	9 (12.5)
	III	25 (34.7)
	IV	12 (16.7)

**Table 4:** Association between comorbidity and prognostic indices.

Comparison	Chi-Square	df	*p* Value	Kappa
GAP vs. TORVAN	32.705	6	<0.001	0.246
GAP vs. Charlson	1.778	4	0.777	−0.052
Charlson vs. TORVAN	5.807	6	0.445	−0.006

**Table 5:** Median survival in the worst prognostic subgroups according to the GAP, TORVAN, and Charlson indices.

Group	*n*	Median Survival (Years)	95% CI (Years)
GAP III	5	4.6	0.0–11.1
TORVAN IV	12	4.6	3.7–5.5
High baseline Charlson	9	2.9	1.2–4.7

Estimates obtained using Kaplan–Meier analysis. Confidence intervals for the GAP III subgroup (n = 5) should be interpreted with caution due to the limited number of patients in this category.

**Table 6:** Cox proportional hazards regression analyses for all-cause mortality.

Variable	HR	95% CI	*p* Value
**Univariable analysis**			
GAP stage	1.99	1.17–3.36	0.011
TORVAN stage	1.43	1.03–1.97	0.031
Charlson index	1.51	0.82–2.77	0.187
FVC, % predicted	0.98	0.96–1.00	0.032
DLCO, % predicted	0.96	0.94–0.98	0.002
**Multivariable analysis**			
GAP stage ^1^	1.43	0.59–3.48	0.428
Charlson index ^1^	1.76	0.95–3.26	0.070
TORVAN stage ^1^	1.21	0.82–1.78	0.341

*HR = hazard ratio; CI = confidence interval; FVC = forced vital capacity; DLCO = diffusing capacity of the lung for carbon monoxide.* ^1^ *Adjusted for age, sex, FVC% predicted, and antifibrotic treatment. DLCO% excluded to avoid collinearity with FVC%*.

**Table 7:** Discriminative performance of Cox regression models.

Model	Harrell’s C-Index	AIC
GAP stage	0.682	216.6
Charlson index	0.692	214.5
TORVAN stage	0.691	214.9

*AIC = Akaike Information Criterion. Higher C-index values indicate better discriminative ability. Lower AIC values indicate a better model fit. A difference in AIC < 2 is considered non-substantial*.

## Data Availability

The original contributions presented in this study are included in the article. Further inquiries can be directed to the corresponding author.

## References

[1] Raghu G., Remy-Jardin M., Myers J.L., Richeldi L., Ryerson C.J., Lederer D.J., Behr J., Cottin V., Danoff S.K., Morell F. (2018). Diagnosis of Idiopathic Pulmonary Fibrosis. An Official ATS/ERS/JRS/ALAT Clinical Practice Guideline. Am. J. Respir. Crit. Care Med..

[2] Raghu G., Remy-Jardin M., Richeldi L., Thomson C.C., Inoue Y., Johkoh T., Kreuter M., Lynch D.A., Maher T.M., Martinez F.J. (2022). Idiopathic Pulmonary Fibrosis (an Update) and Progressive Pulmonary Fibrosis in Adults: An Official ATS/ERS/JRS/ALAT Clinical Practice Guideline. Am. J. Respir. Crit. Care Med..

[3] Gonnelli F., Bonifazi M., Hubbard R. (2024). Mortality trends in idiopathic pulmonary fibrosis in Europe between 2013 and 2018. Eur. Respir. J..

[4] Tran T., Šterclová M., Mogulkoc N., Lewandowska K., Müller V., Hájková M., Kramer M.R., Jovanović D., Tekavec-Trkanjec J., Studnicka M. (2020). The European MultiPartner IPF registry (EMPIRE): Validating long-term prognostic factors in idiopathic pulmonary fibrosis. Respir. Res..

[5] Raghu G., Amatto V.C., Behr J., Stowasser S. (2015). Comorbidities in idiopathic pulmonary fibrosis patients: A systematic literature review. Eur. Respir. J..

[6] Caminati A., Lonati C., Cassandro R., Elia D., Pelosi G., Torre O., Zompatori M., Uslenghi E., Harari S. (2019). Comorbidities in idiopathic pulmonary fibrosis: An underestimated issue. Eur. Respir. Rev..

[7] Kreuter M., Ehlers-Tenenbaum S., Palmowski K., Bruhwyler J., Oltmanns U., Muley T., Heussel C.P., Warth A., Kolb M., Herth F.J.F. (2016). Impact of Comorbidities on Mortality in Patients with Idiopathic Pulmonary Fibrosis. PLoS ONE.

[8] Jovanovic D.M., Šterclová M., Mogulkoc N., Lewandowska K., Müller V., Hájková M., Studnicka M., Tekavec-Trkanjec J., Littnerová S., Vašáková M. (2022). Comorbidity burden and survival in patients with idiopathic pulmonary fibrosis: The EMPIRE registry study. Respir. Res..

[9] Aoki A., Hara Y., Fujii H., Murohashi K., Nagasawa R., Tagami Y., Enomoto T., Matsumoto Y., Masuda M., Watanabe K. (2023). The clinical impact of comorbidities among patients with idiopathic pulmonary fibrosis undergoing anti-fibrotic treatment: A multicenter retrospective observational study. PLoS ONE.

[10] Lee J.H., Park H.J., Kim S., Kim Y.-J., Kim H.C. (2023). Epidemiology and comorbidities in idiopathic pulmonary fibrosis: A nationwide cohort study. BMC Pulm. Med..

[11] Ley B., Ryerson C.J., Vittinghoff E., Ryu J., Tomassetti S., Lee J.S., Poletti V., Buccioli M., Elicker B.M., Jones K.D. (2012). A multidimensional index and staging system for idiopathic pulmonary fibrosis. Ann. Intern. Med..

[12] Charlson M.E., Pompei P., Ales K.L., MacKenzie C.R. (1987). A new method of classifying prognostic comorbidity in longitudinal studies: Development and validation. J. Chronic Dis..

[13] Cottin V., Richeldi L., Kirchgaessler K.U., Schlenker-Herceg R., Quaresma M., Stowasser S., Flaherty K.R. (2020). Efficacy and Safety of Nintedanib in Patients with Idiopathic Pulmonary Fibrosis: Subgroup Analyses by TORVAN Stage. Am. J. Respir. Crit. Care Med..

[14] Simone F., Bocchino M.L., Capitelli L., Del Vecchio K., Laricchiuta A., Scioscia G., Barbaro M.P.F., Lacedonia D. (2020). Torvan Versus Gap: Which Performs Better?. Eur. Respir. J..

[15] Torrisi S.E., Ley B., Kreuter M., Wijsenbeek M., Vittinghoff E., Collard H.R., Vancheri C. (2019). The added value of comorbidities in predicting survival in idiopathic pulmonary fibrosis: A multicentre observational study. Eur. Respir. J..

[16] Podolanczuk A.J., Raghu G. (2024). Idiopathic pulmonary fibrosis mortality: Update on trends in the modern treatment era. Eur. Respir. J..

[17] Sonaglioni A., Caminati A., Elia D., Trevisan R., Zompatori M., Grasso E., Lombardo M., Harari S. (2023). Comparison of clinical scoring to predict mortality risk in mild-to-moderate idiopathic pulmonary fibrosis. Minerva Medica.

[18] Ryerson C.J., Vittinghoff E., Ley B., Lee J.S., Mooney J.J., Jones K.D., Collard H.R. (2014). Predicting Survival Across Chronic Interstitial Lung Disease: The ILD-GAP Model. Chest.

[19] Sun X., Lei S., Zhao H., Guo L., Wang Y. (2024). Mortality-related risk factors of idiopathic pulmonary fibrosis: A systematic review and meta-analysis. J. Thorac. Dis..

[20] Bocchino M., Bruzzese D., Scioscia G., Capitelli L., Tondo P., Rea G., Barbaro M.P.F., Lacedonia D. (2023). Disease stage-related survival in idiopathic pulmonary fibrosis patients treated with nintedanib and pirfenidone: An exploratory study. Respir. Med. Res..

[21] Luppi F., Kalluri M., Faverio P., Kreuter M., Ferrara G. (2021). Idiopathic pulmonary fibrosis beyond the lung: Understanding disease mechanisms to improve diagnosis and management. Respir. Res..

[22] Bargagli E., Bonti V., Ferrari K., Rosi E., Bindi A., Bartolucci M., Chiara M., Voltolini L. (2017). Lung Cancer in Patients with Severe Idiopathic Pulmonary Fibrosis: Critical Aspects. In Vivo.

